# The Epidemiology of Nontuberculous Mycobacteria in Cystic Fibrosis

**DOI:** 10.3390/children12091270

**Published:** 2025-09-22

**Authors:** Aikaterini Sotiropoulou, Ioanna Loukou, Christiana Vliora, Konstantinos Douros, Maria Moustaki

**Affiliations:** 1 Cystic Fibrosis Department, “Agia Sofia” Children’s Hospital, 11527 Athens, Greece; sotirop.kater@gmail.com (A.S.); i.loukou@paidon-agiasofia.gr (I.L.); 2 1st Health Authority of Attica, 11521 Athens, Greece; xristiannavliora@gmail.com; 3 Pediatric Allergy and Respiratory Unit, 3rd Department of Pediatrics, “Attikon” University Hospital, School of Medicine, National and Kapodistrian University of Athens, 12462 Athens, Greece; kdouros@med.uoa.gr

**Keywords:** cystic fibrosis, nontuberculous mycobacteria, *M. intracellulare*, *M. abscessus*, epidemiology

## Abstract

**Highlights:**

**What are the main findings?**

- NTM is a concern for patients with CF even in the era of CFTR modulators
- The prevalence of NTM in CF patients varies considerably among studies making difficult to have a global estimation of the NTM burden in CF

**What is the implication of the main finding?**

- Further multicenter prospective studies are needed to precisely assess the global burden of NTM in CF in the era of CFTR modulators

**Abstract:**

Background: Nontuberculous mycobacteria (NTM) are opportunistic pathogens responsible for chronic pulmonary infections, primarily affecting individuals with underlying conditions such as cystic fibrosis (CF). The aim of this review is to present the epidemiological profile of NTM in CF patients, with a focus on incidence, prevalence, predominant species, and geographic distribution. Methods: The search included cross-sectional, retrospective, and prospective observational studies published in English that reported epidemiological data concerning the isolation and/or infection of individuals with CF by NTM. NTM infection was defined as the isolation of any NTM species at least once per patient. Out of an initial 1120 references identified in PubMed, and following the application of exclusion criteria based on PRISMA guidelines, a total of 78 studies were included. Results: The reported prevalence of NTM in CF patients ranges globally from 0% to 40.9%. This wide variability is attributed to population heterogeneity, study period, and geographical region. Of the studies included, 30 were conducted in Europe and 25 in the U.S.A. *Mycobacterium abscessus* and the *Mycobacterium avium* complex (*MAC*) were the most commonly isolated species, with *MAC* being more prevalent in older individuals. The incidence of NTM pulmonary disease was high, with the majority of cases being associated with *M. abscessus*. Although emerging evidence suggests that CFTR modulator therapy may reduce the risk of NTM isolation and/or disease, current data remain limited. Conclusions: Nontuberculous mycobacteria are significant pathogens in patients with cystic fibrosis, with a negative impact on respiratory health.

## 1. Introduction

Cystic fibrosis (CF) is an autosomal recessive genetic disease [1]. It affects more than 30,000 individuals in the United States and approximately 80,000 people worldwide [2]. The disease is caused by mutations in the CFTR (Cystic Fibrosis Transmembrane Conductance Regulator) gene, located on chromosome 7 [2]. Dysfunction of this gene results in the production of thick, viscous mucus and promotes chronic airway inflammation, which is particularly harmful to the lungs [3,4]. More than 2000 mutations have been identified, which are classified into six categories based on their impact on CFTR function [3].

Respiratory involvement is the most severe manifestation of the disease and represents the leading cause of death or lung transplantation in early adulthood [1]. Morbidity and mortality are mainly due to bronchiectasis, small airway obstruction, and progressive respiratory failure [4]. The most frequently isolated pathogens in CF are *Staphylococcus aureus*, *Pseudomonas aeruginosa* and *Haemophilus influenzae*. Other, less common, and more difficult-to-treat organisms include *Burkholderia cepacia*, *Achromobacter xylosoxidans*, *Stenotrophomonas maltophilia*, and nontuberculous mycobacteria (NTM) [1]. NTM have emerged as significant opportunistic organisms associated with adverse clinical outcomes. They represent a significant challenge in the management of pulmonary disease in CF patients, as it remains difficult to both diagnose and treat effectively [5,6].

Over the past decade, an increase in the detection rates of NTM among CF patients has been observed, prompting intensified surveillance and reporting of these infections. Despite the availability of data from cystic fibrosis registries, the global epidemiological burden of NTM infections in this population remains unclear. The epidemiological landscape of NTM in CF is notably heterogeneous, with substantial variation in prevalence, incidence, age distribution, and geographic trends and there is not a sufficient number of reviews with detailed presentations of NTM epidemiology, especially in pediatric populations.

Based on these observations, the present review aims to analyze the published literature on the epidemiology of NTM infections in patients with cystic fibrosis, with a particular focus on

- Incidence;
- Prevalence;
- Predominant NTM species;
- Geographic distribution.

The ultimate goal is to contribute to a more comprehensive understanding of the epidemiological burden of NTM in the CF population.

## 2. Materials and Methods

### 2.1. Literature Search

A literature search was conducted in the PubMed database in June 2025 (last date 30 June 2025).

Because the review was not registered as a protocol and due to practical resource constraints, the search was limited to PubMed. This decision was made a priori, with the understanding that PubMed has extensive coverage of biomedical literature. The search strategy and eligibility criteria were defined a priori, before the screening process began.

The keywords used to conduct the search were ((mycobacterium) OR (mycobacteria)) AND (cystic fibrosis).

The search included cross-sectional, retrospective, and prospective observational studies that reported epidemiological data concerning the isolation and/or infection of individuals with CF by NTM. NTM infection was defined as the isolation of any NTM species at least once per patient. NTM pulmonary disease was defined following the ATS/ISDA criteria [7,8,9] depending of the year of article publication. It should be mentioned, however, that the ATS/ISDA diagnostic criteria of NTM PD were not different between 2007 and 2020.

The review protocol was not registered in an electronic database. The search strategy and eligibility criteria were defined a priori, before the screening process began.

Data were extracted by two investigators who reviewed the results independently. Any disagreements were resolved by discussion and consensus.

### 2.2. Inclusion and Exclusion Criteria

Studies were included if they met the following inclusion criteria:

- Population: individuals diagnosed with cystic fibrosis.
- Topic: report of the prevalence and/or incidence of NTM infections.
- Study design: prospective or retrospective observational studies, case–control studies, or cross-sectional studies.
- Language: published in English.

The following exclusion criteria were applied:

- Review articles, meta-analyses, case reports, editorials, letters to the editor, and commentaries.
- Studies focusing exclusively on patients with other chronic pulmonary diseases (e.g., bronchiectasis only).
- Studies primarily addressing treatment rather than the epidemiological characteristics of NTM infections.
- Articles lacking sufficient data on the incidence and/or prevalence of NTM infections.

### 2.3. Study Selection

The initial database search yielded 1120 publications from PubMed. These were screened by title and abstract. Of those, 856 articles were excluded due to irrelevance. The remaining 264 records underwent abstract screening and full-text assessment for eligibility. From these, the following articles were excluded for the reasons below:

- Review articles (n = 69);
- Case reports (n = 13);
- Non-English articles (n = 12);
- Articles not reporting epidemiological data (n = 63);
- Articles not related to cystic fibrosis (n = 19);
- Articles not related to NTM (n = 1);
- Not research (editorials, letters to the editor, and commentaries) (n = 9).

**Figure 1:**
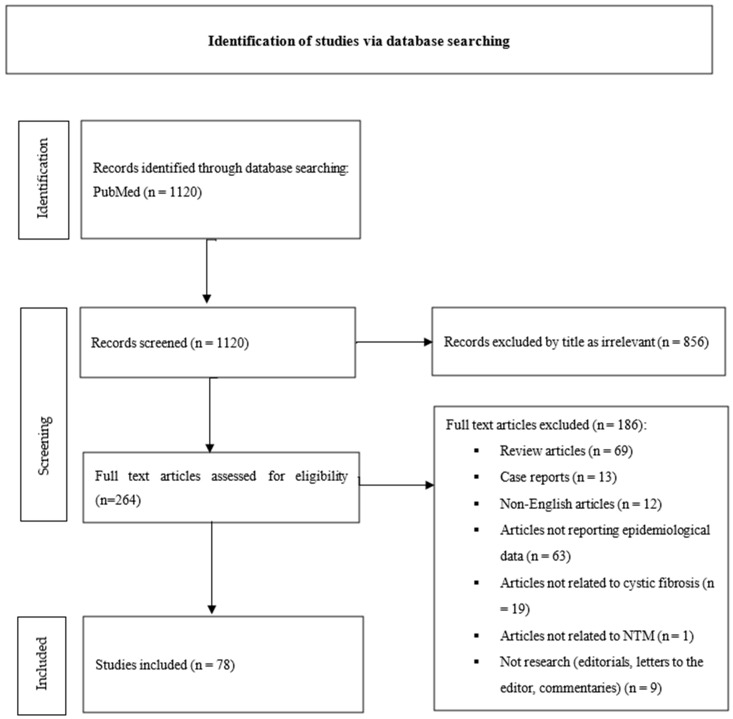
PRISMA flow diagram of study selection.

Finally, 78 studies met the inclusion criteria and were included in the systematic review. The study selection process is illustrated in Figure 1 (PRISMA flow diagram) [10].

### 2.4. Data Analysis

From each study, the following data were extracted: authors, year of publication, country of origin, sample size, age group of participants, detected NTM species, and reported prevalence (annual or periodic) and/or incidence rates.

The extracted data were organized into tables and presented descriptively. The review focused on describing the geographic and age-related distribution of NTM infections among individuals with CF, as well as identifying potential factors contributing to the observed heterogeneity in the findings.

## 3. Results

The reviewed literature distinguishes between annual, period (or overall), and point prevalence of NTM infection. Annual prevalence is defined as the proportion of patients with at least one positive NTM culture within a single calendar year. Period or overall prevalence refers to the proportion of individuals who tested positive for NTM at any point during a follow-up period. In contrast, point prevalence describes the proportion of individuals with a positive culture for NTM at a specific point in time.

Furthermore, according to the ATS 2007 and 2020 criteria, NTM-PD was defined as follows: pulmonary symptoms, nodular or cavitary opacities on chest radiograph, or an HRCT scan that shows multifocal bronchiectasis with multiple small nodules, with appropriate exclusion of other diagnoses; positive microbiological culture results from at least two separate expectorated sputum samples (if the results from the initial sputum samples are non-diagnostic, consider repeat sputum AFB smears and cultures); or positive culture results from at least one bronchial wash or lavage [8,9].

The reported overall prevalence of NTM infections varied considerably across studies, ranging from 0% (Arvind B. et al., 2020 [11], in a cohort of 104 children from India) to 40.9% (Al-Momani H. et al., 2017 [12], in a study of 16 gastrostomy fed patients with CF in the UK).

As it is on the Table 1, in the study by Kilby JM et al., which examined 87 adult cystic fibrosis patients from a single clinic in North Carolina, USA, the prevalence of atypical mycobacterial infection was reported at 20%. The patients who tested positive were older, although no correlation was found with disease severity. Pulmonary function varied widely among patients. Frequent infections included *Pseudomonas aeruginosa*, *Staphylococcus aureus*, *Haemophilus influenzae*, and *Aspergillus* spp. [13].

In a prospective study of 64 adult patients in Seattle, Aitken ML et al. reported a prevalence of NTM infection of 12.5%. The most frequently isolated species was *Mycobacterium avium*. The presence of NTM was not significantly associated with age. Pulmonary function did not differ substantially between NTM-positive and NTM-negative patients, and no significant changes in spirometric values were observed in the NTM-positive group over the following 2–3 years [14].

Bange FC. et al. conducted a retrospective study analyzing samples from 214 adult cystic fibrosis patients followed at a CF clinic in Hanover, Germany, aiming to investigate the potential person-to-person transmission of *Mycobacterium abscessus*. The prevalence of NTM infection in this study was estimated at 7%. The isolated species, in order of frequency, were *M. abscessus*, *Mycobacterium intracellulare*, *Mycobacterium avium*, *Mycobacterium simiae*, *Mycobacterium interjectum*, and *Mycobacterium gordonae*. Each patient’s *M. abscessus* isolate had a unique genotype, suggesting, according to the authors, the absence of person-to-person transmission [15].

Leitritz L. et al. conducted a prospective observational study in Germany involving 91 adult patients, reporting an overall NTM prevalence of 11% for the period 1999–2001 and a point prevalence of 3.3% for the year 1999. The incidence was also calculated at 8%. The most frequently isolated species were *M. avium* and *M. intracellulare*. In this study, patients with cystic fibrosis tended to have a higher frequency of NTM isolation compared to non-CF patients. However, neither the prevalence nor the rate of NTM clearance differed significantly between the two groups [16].

Girón RM et al. conducted a prospective study of 28 adult cystic fibrosis patients followed at the Hospital Universitario de la Princesa in Madrid, Spain, between 1997 and 2001. The prevalence of NTM infection was estimated at approximately 25%, with the most frequently isolated species being *M. abscessus*, *M. avium* complex, and *M. simiae* [17].

Chalermskulrat W. et al. conducted a retrospective study in Ohio examining the frequency of NTM isolation in patients with end-stage cystic fibrosis, before and after lung transplantation, as well as its impact on transplant outcomes. The retrospective analysis included 146 lung transplant recipients and 31 transplant candidates. The frequency of NTM isolation prior to transplantation was 19.7%, while post-transplant isolation was 13.7% of cases. However, invasive NTM infection following transplantation was rare (3.4%). Among NTM-positive end-stage CF patients, there were no significant differences compared to NTM-negative patients in terms of age, FVC, FEV1, or co-infection with other bacterial pathogens such as *Pseudomonas aeruginosa*, *Burkholderia cepacia*, or *Staphylococcus aureus* (*p* > 0.2 for all). The most commonly isolated NTM species was *M. avium* (45%), followed by *M. abscessus* (41%). Other species included *M. gordonae* (7%) and *M. fortuitum* (3%). The majority of patients with NTM pulmonary disease prior to transplantation were infected with *M. abscessus*. Pre-transplant NTM isolation was significantly associated with increased risk of developing NTM pulmonary disease after transplantation (OR: 6.13, *p* = 0.001), with *M. abscessus* being the only species with a statistically significant correlation (OR: 7.45, *p* = 0.005). No such association was found for other NTM species. The authors noted that patients with a positive NTM culture prior to transplantation were five times more likely to have recurrent NTM isolation post-transplant compared to those who were NTM-negative (*p* = 0.008). Among those with post-transplant NTM recurrence, 71% isolated the same species as before transplantation. The most frequently isolated species after transplantation were *M. avium* (39%), *M. gordonae* (30%), and *M. abscessus* (13%), followed by *M. kansasii* (9%), *M. szulgai* (4%), and *M. fortuitum* (4%). Only a few species were responsible for post-transplant pulmonary disease, primarily *M. abscessus* and *M. szulgai*. While NTM disease led to significant morbidity in some post-transplant patients, it generally responded well to treatment and did not adversely affect transplant outcomes. According to the authors, pre-transplant NTM isolation should not be considered a contraindication for lung transplantation in CF patients, although it may indicate an elevated risk of recurrence [18].

The cross-sectional retrospective study of Paschoal IA. et al. used data from 54 adult patients with CF attending the Outpatient Chest Disease Clinic in São Paulo, Brazil and aimed to describe the clinical characteristics and microbiological profile of adult CF population. In this study the prevalence of NTM was 11%. None of these patients received treatment, as they did not meet the ATS criteria for NTM-PD [19].

Coolen N. et al. conducted a study at the Pulmonary Department at Cochin University Hospital in Paris between 2006 and 2010, aiming to investigate the association between azithromycin use in CF patients and the isolation of NTM, as well as macrolide susceptibility. The study included 347 adult patients, with the overall prevalence of NTM infection estimated at 12.4%. The most frequently isolated species were the *MABSC* (58.5%) and the *Mycobacterium avium*/*intracellulare* complex (48.7%), followed by *Mycobacterium fortuitum*, *Mycobacterium simiae*, and *Mycobacterium kansasii* (12.2%). The prevalence of NTM pulmonary disease was also calculated at 9.5%, with *M. abscessus* being more frequently observed in patients who met ATS diagnostic criteria (*p* < 0.05). Chronic colonization with *Aspergillus fumigatus* and *Haemophilus influenzae* was significantly more common among NTM-infected patients, especially those meeting ATS criteria, whereas *S. aureus* colonization was also more frequent in NTM-positive patients, though the difference was not statistically significant in the ATS-positive subgroup. Regarding azithromycin use, the authors concluded that it may exert a protective effect against NTM infection in adult patients with CF [20].

Al-Momani H. et al. conducted a study including 16 PEG-tube fed CF patients who were attending a CF Center in Newcastle, UK. Following an overnight fast, gastric juice and sputum samples were collected from the patients, and PEG tubes that were replaced or removed as part of routine care were also retrieved. The prevalence of NTM isolation in this population was 43%, with *MABSC* being the only species identified. According to the authors, this was the first identification of *MABSC* in a gastric sample, leading to the conclusion that gastric juice and PEG tubes may represent a potential source of *MABSC* isolation in CF patients [12].

In the study conducted by Sherrard LJ et al. at the Prince Charles Hospital in Queensland, Australia, data from 434 adult CF patients were retrospectively analyzed over the period 2001–2013 to investigate the incidence and risk factors for the isolation of NTM. The overall prevalence of NTM infection was 14%, with significantly higher rates observed among residents of tropical regions (27%) compared to those living in subtropical areas (11%) (*p* = 0.001). *MABSC* was the most frequently isolated species (45%) and was associated with the vast majority (92%) of NTM-PD cases. The second most common species identified was *M. intracellulare* (32%). A statistically significant association was found between *M. abscessus* isolation and geographic region (*p* = 0.02), with higher detection rates among patients from tropical areas (14%) compared to subtropical regions (6%). In contrast, no significant geographic association was noted for *M. intracellulare* (9% vs. 3%, *p* = 0.09). Furthermore, the prevalence of NTM-PD was significantly higher in tropical residents (20%) than in those from subtropical regions (4%) (*p* < 0.001) [21].

The retrospective study by Fernández-Caso et al. included 92 adult CF patients attending a hospital in Madrid. The prevalence of NTM infection was 30.4%, and *M. avium* was the most frequently isolated species, followed by *M. abscessus*. Colonization with *M. abscessus* was significantly associated with age under 30 years [22].

Richter WJ et al. included 254 CF patients over the age of 18 in a retrospective cohort study to investigate whether vitamin D deficiency is a risk factor for NTM infection. The overall prevalence of NTM infection in the cohort was 19.7%. *M. avium* was the most frequently isolated species (58%), followed by *M. abscessus*/*chelonae* (36%). Patients who developed NTM infection had higher FEV1 values and were less frequently infected with *P. aeruginosa*. Vitamin D deficiency was associated with an increased risk of NTM infection in adult CF patients. Deficiency was more common among those with positive NTM cultures compared to those with negative cultures (35.1% vs. 22.1%, *p* < 0.01). Furthermore, vitamin D levels measured prior to positive NTM cultures were significantly lower (mean 25.7 ng/mL) than those measured before negative cultures (29.1 ng/mL, *p* = 0.03). Persistent vitamin D deficiency, defined as two or more consecutive low-level measurements, was also more frequent in patients with NTM infection (28%) compared to those who remained culture-negative (15.7%) [23].

In the retrospective study by Wyrostkiewicz D. et al., which included 151 CF patients who were hospitalized due to pulmonary exacerbation in a CF center in Poland between 2010 and 2020, the prevalence of NTM infection was 7%. The majority of these patients met the diagnostic criteria for NTM-PD. The most frequently isolated species was *M. avium* (55%), followed by *M. chimaera* (18%), *M. kansasii*, *M. abscessus*, and *M. lentiflavum*. Reported spirometry values were FVC 73.8% (SD 16.7%), FEV1 58.6% (SD 20.6%), and FEV1/FVC 70.1 (SD 14.33). Among NTM-positive patients, 63% were co-colonized with *P. aeruginosa*, 55% with *S. aureus*, and 45% with *A. fumigatus*. Additionally, 46% were concurrently diagnosed with ABPA [24].

Gross JE et al. investigated retrospectively the possibility of healthcare-associated transmission of NTM among CF patients receiving care at the Colorado Adult Cystic Fibrosis Program. This study included 507 patients and prevalence of NTM infections was estimated at 32.5%. While the study did not provide evidence of frequent healthcare-associated transmission of NTM, it highlighted the need for stricter methods to clarify the origin of such infections [25].

The retrospective observational study by Mianowski L. et al. involved 198 adult patients followed at the CF Center at the University Hospital of Lyon, France. The study analyzed sputum cultures for three years prior to and one year following initiation of the triple combination therapy elexacaftor–tezacaftor–ivacaftor (ETI). A significant reduction in the prevalence of colonization by common CF pathogens was observed, decreasing from 99% to 90% after one year of ETI treatment. Furthermore, the prevalence of patients positive for NTM in the three years prior to CFTR modulator therapy was 7.2%, 8.7%, and 10%, respectively, but declined significantly to 1.7% after treatment (*p* < 0.001) [26].

Örlős Z. et al., in their retrospective case–control study in Hungary, used data from 232 adults in the Hungarian CF Registry and reported an increasing overall prevalence of NTM infection, rising from 4.7% in 2020 to 12.9% in 2022. The most commonly isolated NTM species were *MAC* (41.0%), *MABSC* (38.5%), and *Mycobacterium xenopi* (15.4%). FEV1 values were significantly lower in the NTM-positive group at the time of diagnosis and 1–2 years prior to NTM detection (48.0% vs. 64.9%, *p* < 0.01). However, the rate of FEV1 decline (ΔFEV1) did not differ significantly between the NTM-positive and NTM-negative groups, suggesting that reduced lung function may be a potential predictive factor for NTM infection, rather than a consequence of accelerated pulmonary deterioration due to infection. The study highlights the importance of intensified screening for NTM, particularly in patients with FEV1 ≤ 50%. Among NTM-positive patients, 67% met the 2007 ATS/IDSA diagnostic criteria for NTM-PD. All patients infected with *MABSC* had pulmonary disease, whereas 69% of those with *MAC* infection developed NTM-PD [27].

Gross JE et al. conducted a study in Texas to investigate potential healthcare-associated transmission or acquisition of NTM at the same CF center. The study included data from 294 adult patients followed between 2013 and 2018, reporting a prevalence of NTM infection of 24.1%. Among those infected, 70.4% were found to have *M. abscessus*, although the proportions of other NTM species were not specified. The authors concluded that in-hospital transmission (whether patient-to-patient or via shared equipment) appeared to be rare at this center. Instead, patients are likely infected through multiple transmission routes outside the healthcare setting [28].

As it is on Table 2, Fauroux B. et al. conducted a prospective study of 106 children with cystic fibrosis and estimated an NTM infection prevalence of 6.6%, an incidence of positive cultures at 2.3%, and a prevalence of NTM pulmonary disease at 1.9% [29].

The study by Esther, Jr. et al. is a retrospective analysis of a pediatric population under 12 years of age, based on 190 patients’ data from the Cystic Fibrosis Foundation Registry (CFFR) and 114 patients from a bronchoscopy database in North Carolina, USA. According to the bronchoscopy data, the mean annual prevalence of NTM infections was 4.3% per year (range 1.2–7.0%), with an overall prevalence of 6.1% for the period 1999–2002. Data from the CFFR showed an overall prevalence of 3.9% for the period 1993–2002, with a mean annual prevalence of 2% per year (range 0.9–4.1%). Among NTM-positive patients, 59% met the American Thoracic Society (ATS) microbiological criteria for NTM pulmonary disease, with no significant differences in age or sex compared to those who did not meet the criteria. *Mycobacterium abscessus* was more frequently isolated in this group (*p* < 0.05). Of those who received treatment, 30% achieved microbiological clearance, defined as three consecutive negative cultures. Clearance was more common in *Mycobacterium avium* infections (three out of four cases) than in *M. abscessus* (one out of four), though this difference was not statistically significant (*p* = 0.48). Patients meeting ATS criteria had a significantly greater annual decline in FEV1 (−4.9 ± 1.4%) compared to those who did not (−2.5 ± 1.4%, *p* < 0.035). A trend for improvement in the rate of FEV1 decline was observed after treatment (1.1 ± 5.1%) compared to the rate before treatment (−7.1 ± 5.6%, *p* = 0.075). This study observed a greater decline in lung function (FEV1%) in children who met the ATS criteria for NTM infection before the age of 12. Although an association with more rapid pulmonary deterioration was identified, causality could not be established due to limitations such as small sample size, unknown timing of infection onset in some cases, and confounding effects of chronic macrolide therapy, which independently affects FEV1. While a trend toward improvement was noted in treated patients, only half achieved microbiological clearance. These findings support the potential direct impact of NTM infection on the decline of lung function in cystic fibrosis patients [30].

In the prospective observational study by Radhakrishnan DK. et al., which included patients aged 6–18 years attending a CF Center in Toronto, Canada, the point prevalence of NTM infection for the year 2004 was estimated at 6.1%. The most commonly isolated NTM species were *M. avium* and *M. abscessus*. Comparison between NTM-positive and NTM-negative groups revealed no significant differences in mean age of infection or FEV1 values, although all NTM-positive patients were over 12 years of age. Among those with NTM-positive cultures, 50% were also colonized with *Aspergillus fumigatus* and 50% with *Staphylococcus aureus* [31].

Cândido PH et al. studied 129 pediatric CF patients followed at a referral center in Rio de Janeiro, Brazil and reported a prevalence of NTM infection of 7.75%. The aim of this study was the molecular identification of NTM species and the analysis of their antimicrobial susceptibility and their genetic diversity. Most of the isolated organisms exhibited multidrug resistance, and the authors emphasized the need for systematic and regular screening for NTM infection or colonization in CF patients, even in countries with a high burden of tuberculosis [32].

In the retrospective study of a pediatric CF population conducted by Satana D. et al. in Turkey, the overall prevalence was found to be 3.07%. In this study, *M. abscessus* was the most frequently isolated species (60.9%), followed by *M. lentiflavum* (39.1%). Co-infection with *S. aureus* was observed in 21.7% of cases, while the rate of co-infection with *P. aeruginosa* was higher, at 34.7% [33].

In the retrospective study by Bouso JM et al., conducted in Florida, the authors aimed to investigate whether residential proximity to water sources was associated with the acquisition of NTM among 65 children with CF. The prevalence of NTM infection was found to be 32.3%. Among those with positive cultures, 66.7% were positive for *MAC*, and 23.8% for *MABSC*. Geographic variation in NTM species distribution was observed: in the Pensacola center, 100% of positive cases involved *MAC*, while in the Orlando center, 58.8% were *MAC* and 41.2% *MABSC*. The study also found that children residing within 500 m of a water source were 9.4 times more likely to acquire NTM. Additionally, a history of *A. fumigatus* infection (*p* = 0.011) and recent infection with *P. aeruginosa* (*p* = 0.007) were significantly associated with a positive NTM culture [34].

In their prospective observational study, Ahmed MI et al. investigated the utility of annual sputum induction in children with CF who do not spontaneously expectorate, aiming to enable the early detection of NTM. The study was conducted at a CF Center in Leicester City between 2012 and 2016 and included 42 children aged 5–17 years. Over the five-year follow-up period, the incidence of NTM infection was 14%. The study concluded that annual induced sputum collection is a safe and effective method for the early detection of NTM infections in non-expectorating children with CF [35].

In the retrospective pediatric study by Gardner AI et al., using data from the UK CF Registry for the period 2010–2015, the overall prevalence of NTM infection was 5.4%. The annual prevalence of patients with positive NTM cultures increased progressively from 1.3% in 2010 to 3.8% in 2015. Children with NTM isolation were significantly older (*p* = 2.2 × 10^−16^), more likely to be colonized with *P. aeruginosa* (45% vs. 30%, *p* = 0.013), and more likely to have ABPA (17% vs. 4.7%, *p* = 2.2 × 10^−16^). However, there was no statistically significant difference in FEV_1_ between NTM-positive and NTM-negative individuals (81.3% vs. 86.4%, *p* = 0.286). Between 2011 and 2013, approximately 40% of NTM-positive cases (around 26 cases per year) represented new infections. In both 2014 and 2015, *M. abscessus* accounted for the majority of identified infections (51.4% and 35.3%, respectively), followed by *M. avium* (11.4% and 8.3%, respectively) [36].

In the retrospective study by Yan J. et al., which included 99 children attending the Royal Children’s Hospital in Melbourne, the recorded prevalence of NTM infection was 11%. The prevalence of *M. abscessus* infection specifically was estimated at 6.7% [37].

Arvind B. et al. conducted a retrospective study at a tertiary care center in northern India, involving 104 children with CF experiencing respiratory deterioration during the period 2013–2015, and recorded no cases of NTM isolation [11].

In the study by Hughes DA et al., which analyzed data from 567 children at the Royal Brompton Hospital CF Center in London, the prevalence of NTM infection was found to be 10.4%. The most frequently isolated species was *MABSC,* followed by *MAC*. The authors did not identify any association with age. Among patients with *MAC*, 43% received treatment, and infection was eradicated in 67% of those cases. Although FEV_1_ was lower in treated *MAC* patients, the difference was not statistically significant. Among patients with *MABSC*, 76% received treatment, and eradication was achieved in 65%. The most common co-infection during the first year of treatment was *A. fumigatus*, found in 78% of *MAC* cases and 54% of *MABSC* cases [38].

Abidin NZ. et al. used data from 4687 patients aged ≤16 years from the UK CF Registry in a retrospective study and reported an overall prevalence of NTM infection of 6.5%. The most commonly isolated species were *M. abscessus* (58.1%) and MAC (30.4%). The annual prevalence remained stable between 2016 and 2018, at 3.5%, 3.1%, and 3.6%, respectively, with similar trends observed for both *M. abscessus* and *MAC*. Higher possibility of NTM isolation was associated with *P. aeruginosa* infection, ABPA, older age, and lower FEV_1_ values [39].

Singh J. et al. analyzed data from 419 patients under the age of 18, who were followed at a major CF center in Australia between 2002 and 2019, aiming to assess annual trends in the incidence and prevalence of respiratory pathogens across different age groups. The overall prevalence of NTM infection was recorded at 0.72%, with an incidence of 3.7%. Among the nine pathogens studied, NTM had the lowest incidence and prevalence. However, a non-significant upward trend was observed over time, except in the 6–11-year age group, where the increase reached statistical significance [40].

As it is on Table 3, Mulherin D. et al., in a prospective study conducted in Dublin involving 43 patients with CF, aimed to investigate the delayed cutaneous hypersensitivity to NTM and the presence of mycobacteria in sputum samples. The study reported a prevalence of 2.3% [41].

In a prospective study conducted in Sweden by Hjelte L. et al., which included 54 patients aged 3 to 67 years, the prevalence of NTM infection was 9.3%. The cases were associated with clinical deterioration and a positive response to treatment [42].

In the study by Hjelt K. et al., which included data from 185 patients over the age of 2 followed at the Danish Cystic Fibrosis Center, a point prevalence of 1.6% for NTM infection was recorded [43].

In the study by Torrens JK et al., a retrospective case–control study using data from 372 pediatric and adult patients from cystic fibrosis clinics in Leeds, UK, the overall prevalence of NTM infections between 1989 and 1994 was estimated to be 3.8%. The most commonly isolated NTM species were *Mycobacterium fortuitum*, *Mycobacterium avium* complex, and *Mycobacterium chelonae*. Pulmonary function, measured using forced vital capacity (FVC) and forced expiratory volume in one second (FEV1), showed no significant difference (*p* = 0.93 and *p* = 0.65, respectively) [44].

In the prospective study by Oliver A. et al., involving 37 pediatric and adult cystic fibrosis patients from a CF clinic in Spain in the year 2000, an overall NTM prevalence of 16.1% was reported [45].

In the prospective study by Sermet-Gaudelus I. et al. conducted in France, the authors analyzed data from 296 cystic fibrosis patients, reporting an NTM infection prevalence of 9.8%. *Mycobacterium abscessus* was the most frequently isolated species. The mean age at first positive culture for *M. abscessus* was similar to that of the overall study population (11.9 years), but younger than that of patients positive for *Mycobacterium avium* complex (mean age 17.5 years). Pulmonary function showed considerable variability [46].

A prospective, cross-sectional study was conducted in 2002 across CF centers in the United States, involving 986 patients over the age of 10. In the study by Olivier KN. et al., the overall prevalence of NTM infection was 13%, with *Mycobacterium avium* complex (72%) and *Mycobacterium abscessus* (16%) being the most commonly isolated species. 20% of patients with positive cultures met the American Thoracic Society’s microbiological criteria for NTM pulmonary disease. The study showed that, compared to culture-negative individuals, NTM-positive patients were older by an average of 4 years and had a higher FEV1 by six percentage points, a 10% lower prevalence of *Pseudomonas aeruginosa*, and a 12% higher prevalence of *Staphylococcus aureus* [47].

Devine M. et al., in a study conducted in Northern Ireland involving 66 adults and 116 children, examined the presence of mycobacterial DNA in sputum samples from cystic fibrosis patients. The prevalence was estimated at 0.9% for children and 3.0% for adults [48].

In the study by Mussaffi H. et al., which included 139 CF patients from Israel, the prevalence of NTM infection was 8.6%, while the prevalence of NTM pulmonary disease was 4.3%. Among those with positive cultures, 50% met the diagnostic criteria for pulmonary disease. *M. abscessus* was the most frequently isolated species. The majority of NTM-PD patients also had allergic bronchopulmonary aspergillosis (ABPA). Notably, most individuals with NTM-PD were under 18 years of age and had chronic *P. aeruginosa* infection [49].

In 2000, Pierre-Audigier C. et al. conducted a multicenter prospective study including 385 patients between the ages of 1 and 24 attending three cystic fibrosis centers in the Paris area. The overall prevalence of NTM infection was estimated at 8.1%. The most frequently isolated mycobacterial species were *M. abscessus* (39.4%), *MAC* (21.2%), and *M. gordonae* (18.2%). Other nontuberculous mycobacteria (21.2%) isolated from sputum cultures included *M. kansasii*, *M. chelonae*, *M. lentiflavum*, *M. scrofulaceum*, *M. szulgai*, and *M. xenopi*. Among the NTM-positive patients, 51.6% met the ATS criteria for NTM pulmonary disease, with the majority of these cases associated with *M. abscessus*. This study highlighted significant differences in NTM prevalence across age groups. The overall prevalence remained relatively stable (~5%) among patients aged 1–14 years but increased sharply to 14.9% in the 15–24 age group (*p* < 0.001). *M. abscessus* was isolated across all age groups (mean age 14.1 years), whereas *MAC* appeared exclusively in patients older than 15 years (mean age 17.3 years), with a significantly higher prevalence in older patients (0% in those aged 1–14 years vs. 5.2% in those aged 15–24 years, *p* < 0.001). *M. gordonae* was primarily isolated in the younger group, with prevalence rates of 2.0% and 0.7% in the 1–14 and 15–24 age groups, respectively (*p*-value not significant). Other species, such as *M. kansasii*, followed a pattern similar to *MAC*, with prevalence rates of 0.4% and 4.4% in the 1–14 and 15–24 age groups, respectively (*p* < 0.01). Overall, *M. abscessus* and *M. gordonae* were predominant in patients aged 1–14 years, whereas *M. abscessus* and *MAC* were most frequently isolated in those aged 15–24 years [50].

The study by Ferroni A. et al. analyzed sputum samples from 289 pediatric and adult CF patients, with the overall prevalence of NTM-positive cultures estimated at 11%. The most frequently isolated species were *Mycobacterium abscessus* (72%), *M. gordonae* (11%), *M. avium* (5%), and *M. chelonae* (5%) [51].

In the study by Valenza G. et al., which included 90 both pediatric and adult patients from a single CF center in Germany, the prevalence of NTM was reported to be 13.3% [52].

The study by Levy Ι. et al., a multicenter, cross-sectional, prospective study that used medical records of 186 pediatric and adult patients from six CF centers across Israel, reported 22.6% overall prevalence of NTM infection with geographically variable distribution. Higher rates were observed in central and southern Israel compared to the north. The most commonly isolated NTM species were *M. simiae* (40.5%), *M. abscessus* (31.0%), and *MAC* (14.3%). Patients with *M. abscessus* were younger (mean age 18.46 ± 6.42 years) compared to those with other NTM species (26.79 ± 11.63 years, *p* < 0.05) and more frequently co-infected with *S. maltophilia* (15.4% vs. 0%, *p* < 0.05). According to the 1997 ATS criteria, 6.5% of patients fulfilled the diagnostic criteria for NTM pulmonary disease, and 10.8% according to the 2007 ATS guidelines. *M. simiae* was isolated in half of the patients who met the ATS criteria. Additionally, NTM-positive patients were older on average (by 4.8 years, *p* = 0.014), had lower FEV1 values (by 14.5 L/s, *p* = 0.0001), and showed higher rates of *Pseudomonas aeruginosa* (95.2% vs. 65.3%) and *Aspergillus* spp. (66.7% vs. 21.5%) in sputum cultures compared to control patients [53].

The multicenter prospective observational study by Roux AL. et al., which included 1582 cystic fibrosis patients (both children and adults) from 31 CF centers across France, reported an overall NTM prevalence of 6.6%. The most frequently isolated species were *MABSC* (*Mycobacterium abscessus* Complex) and *MAC*. The highest prevalence was observed in the 11–15-year age group (10.4%), with *MABSC* being the predominant species across all ages, particularly in the 11–15-year group (5.8%). In contrast, *MAC* was not isolated in patients under the age of 9 and peaked in individuals over 25 years old (2.2%). In patients up to 25 years of age, *MABSC* was more frequently isolated than *MAC* (3.7% vs. 1.2%), with a statistically significant difference observed in the 11–15-year age group (*p* = 0.02). The highest prevalence was recorded in the greater Paris area (9.6%), while the lowest was in eastern France (3.7%). In the Paris region, *MABSC* was significantly more prevalent than *MAC* (5.1% vs. 1.0%, *p* = 0.007), and this region accounted for 42% of all *MABSC*-positive cases. Among patients who tested positive for NTM, 54.8% met the ATS criteria for NTM-PD, corresponding to a prevalence of 3.6% in the overall study population. The prevalence of patients meeting the ATS bacteriological criteria for *MABSC* and *MAC* pulmonary disease was 2.5% and 1.1%, respectively [54].

In the retrospective observational study by Seddon P. et al., which included 7122 children and adults from CF centers across the United Kingdom, the prevalence of NTM infection was estimated at 5% among adults and 3.3% among children, with an overall prevalence of 4.2%. The most frequently isolated NTM pathogens were the rapidly growing *MABSC*, found in 62% of NTM-positive adults and 68% of NTM-positive children, followed by *MAC*, identified in 28% of adults and 27% of children. A notable geographical variation in prevalence was observed, increasing from the northwest to the southeast, with the lowest rate recorded in Northern Ireland (1.9%) and the highest in Southeast England (7.5%) [55].

In the study by Esther CR Jr et al., which analyzed data from 1216 pediatric and adult patients registered in the PortCF database at the University of North Carolina, USA, the overall prevalence of NTM infection was estimated at 13.7% and the mean annual prevalence at 10.8%. Among the identified isolates, *M. avium* accounted for 59% and *M. abscessus* for 41%. Prevalence was slightly higher among older CF patients (13.2 ± 3.8% in individuals aged 40+), compared to adolescents and young adults (9.9 ± 2.6% for ages 14–39, *p* <0.05). The authors highlighted that while these findings are consistent with previous reports, direct comparisons between age groups are limited by differences in sampling methods: AFB cultures in young children (≤5 years) were predominantly obtained via bronchoalveolar lavage, whereas older patients provided expectorated sputum samples. The number of patients with at least one positive NTM culture increased over the study period (2000–2007), with an annual rise of 0.7 ± 0.2% in the overall cohort (*p* < 0.01). A significant association between pulmonary function and age was noted, with an annual decline in predicted FEV1 of −1.64%. Patients with *M. abscessus* infection experienced an additional annual decrease of −0.78%, resulting in a total decline of −2.42% per year (*p* = 0.02). Those with chronic infection by other NTM species showed an intermediate rate of FEV1 decline, although this was not statistically significant. Overall, patients chronically infected with NTM exhibited a significantly greater annual decline in predicted FEV1 (−2.33%, *p* < 0.01) compared to NTM-negative patients. Co-culture analysis revealed that samples positive for NTM were more frequently associated with *S. maltophilia* (18.2% vs. 8.4%, *p* < 0.01) and *A. fumigatus* (13.9% vs. 7.2%, *p* < 0.01), while *P. aeruginosa* was less frequently isolated in NTM-positive cultures (50.8% vs. 57.9%, *p* < 0.01) [56].

In the retrospective case–control study by Binder AM et al., which used 5403 patients’ data recorded in the Cystic Fibrosis Patient Registry (CFPR), the point prevalence of NTM infection in 2011 was estimated at 4%. Among NTM-positive patients, 64% had MAC infection, while 36% had *M. abscessus*. Patients with *MAC* infection exhibited similar characteristics to control subjects in terms of age (mean: 25 years) and mean FEV1% (73% vs. 69%). However, individuals with *M. abscessus* infection had significantly higher FEV1 values compared to controls (76% vs. 69%; OR = 1.1 per 5% increase, *p* < 0.05). MAC-positive patients were significantly more likely to be colonized with *S. maltophilia* (OR = 1.6, *p* < 0.05) and *Aspergillus* spp. (OR = 1.7, *p* < 0.001) and less likely to be colonized with *P. aeruginosa* (OR = 0.7, *p* < 0.05) in comparison to NTM-negative individuals. Similarly, *M. abscessus* cases were significantly more likely to be colonized with *S. maltophilia* (OR = 2.9, *p* < 0.001) and *Aspergillus* spp. (OR = 3.1, *p* < 0.001) compared to controls. These findings suggest a possible association between specific microbial colonizations and the development of NTM infection in individuals with cystic fibrosis [57].

The study by Qvist T. et al. aimed to evaluate the specificity of the urine lipoarabinomannan (LAM) strip test in individuals with NTM infections. The study was conducted using samples from 198 CF patients in Denmark, a country with a high incidence of NTM and minimal tuberculosis exposure. The prevalence of NTM infection was estimated at 12%, and 83% of NTM-positive patients met the criteria for NTM pulmonary disease. A limitation of the study was that 35% of patients were excluded due to unavailable urine samples [58].

Martiniano SL. et al. conducted a retrospective study of 650 children and adults with CF followed at the Colorado CF Center between 2000 and 2010. The overall prevalence of NTM infection was found to be 14.8%, 9.6% in children and 31.7% in adults. The most frequently isolated species was *MAC* (75% in children, 69% in adults), followed by *M. abscessus* (21% in children, 27% in adults). NTM-positive adults were younger than NTM-negative adults, but no age-related differences were observed between species. NTM-positive adults had lower FEV1 values than children but higher than NTM-negative adults. A total of 38.5% of NTM-positive patients met the criteria for active pulmonary disease, with no predominance of either *MAC* or *M. abscessus*. Those patients had lower FEV1 and a greater rate of decline. *S. aureus* was the most commonly co-isolated bacteria among NTM-positive patients, while *S. maltophilia* and *A. fumigatus* were found more frequently compared to NTM-negative individuals. Moreover, NTM-positive adults more frequently isolated *P. aeruginosa* than children and *S. aureus* than NTM-negative patients. Approximately one-quarter of patients with a single positive culture tested negative in follow-up sputum cultures. Additionally, a subgroup of patients showed persistent culture positivity without clinical signs of active disease, supporting the concept of subclinical infection. Despite the high failure rate of eradication therapy, lung function stabilized after treatment initiation. The authors emphasized the need for long-term prospective studies to identify early markers predictive of disease progression and to guide intervention [59].

Adjemian J. et al., in a retrospective study using data from the U.S. CF Patient Registry involving 10,527 patients over 12 years, estimated an overall NTM infection prevalence of 14%. The prevalence of NTM infection showed significant geographic variation across the United States, ranging from 0% to 28%. The highest prevalence was observed in Alaska, South Florida, Wisconsin, and Louisiana, where *M. abscessus* predominated, whereas *MAC* was more frequently isolated in Arizona [60].

The retrospective study by Raidt L. et al., which included 94 patients who attended two CF centers in Münster, Germany, reported that no NTM isolates were detected in 2001. However, by 2011, the prevalence of NTM infection had increased to 7.4% and M.abscessus was the most frequently identified species [61].

Bar-On O. et al. conducted a retrospective observational study of 110 pediatric and adult patients at a CF center in Israel through medical record review. The annual incidence of NTM infection increased from 0% in 2002 to 9% in 2011 (*p* < 0.001) and the annual prevalence rose from 5% in 2003 to 14.5% in 2011 (*p* = 0.05). *M. abscessus* was identified in 46% of cases and *MAC* was identified in 24%. The incidence of NTM pulmonary disease peaked in 2009 (11.9%) and declined thereafter to 5.5% in 2011, with *M. abscessus* being more frequently associated with NTM-PD (69%, *p* = 0.0004). Patients with NTM were more likely to have chronic *P. aeruginosa* infection (*p* = 0.06), and there was a strong association between NTM infection and airway colonization by Aspergillus species (*p* = 0.003), as well as with allergic bronchopulmonary aspergillosis (ABPA) (*p* = 0.01). FEV1 values were similar between patients with and without NTM infection [62].

The prospective study by Phelippeau M. et al. aimed to determine the frequency of Mycobacterium lentiflavum in CF patients in Marseille. The overall prevalence of NTM infection was 7.1%. *M. abscessus* accounted for 48% of cases, followed by the *Mycobacterium avium* complex (32%) and *M. lentiflavum* (24%) [63].

The retrospective observational study by Qvist T. et al. based on data from CF registries and microbiological databases in Denmark, Norway, and Sweden analyzed a cohort of 1411 pediatric and adult patients over a 13-year period (2000–2012) and reported an overall NTM prevalence of 11%. The study documented a gradual increase in the annual prevalence of infections caused by *MABSC* and *MAC*, rising from 1.7% in 2000 to 4.1% in 2012. Geographic differences were observed in incidence, ranging from 3% in Denmark to 28% in Sweden, with *MABSC* predominating in Denmark and *MAC* in Norway and Sweden. *MABSC* was isolated in 45% of cases and *MAC* in 32%, while 11% of patients had co-infection with both species. The mean age at first positive NTM culture was 19 years, and 45% of patients had concurrent chronic infection, most commonly with *P. aeruginosa* (34%). *MABSC* was acquired at a younger age than *MAC* (median age 17 vs. 22 years, *p* < 0.01). Approximately 80% of NTM-positive patients met the ATS/IDSA diagnostic criteria. Most patients infected with *MABSC* acquired the infection before the age of 20 and were more likely to receive treatment compared to those with *MAC* (72% vs. 44%, *p* = 0.02). Despite treatment, only 39% of cases achieved culture conversion. Patients with *MABSC* showed higher rates of treatment failure and advanced lung disease, although these differences did not reach statistical significance [64].

Kopp BT. et al. conducted a study aimed at investigating potential geographic differences of the demographic characteristics of CF patients across the United States. Using data from 30,896 patients in the Cystic Fibrosis Foundation Patient Registry (CFFPR), the authors estimated an overall NTM infection prevalence of 8.1%. The proportion of patients with at least one positive culture was higher in the western (9.6%) and southern (10.0%) regions of the country [65].

In the retrospective study by Campos-Herrero MI. et al. which involved 44 pediatric and adult cystic fibrosis patients monitored at a clinical center in Gran Canaria, the overall prevalence of NTM infection over the period 2002–2012 was found to be 40.9%. The mean annual prevalence was estimated at 14.1%, with rates of 18.5% in patients under 15 years and 10.4% in older individuals. Annual prevalence declined from 33.3% in 2002 to 12.5% in 2005, remaining relatively stable thereafter. The annual incidence rate was highly variable, ranging from 0% to 14.3%. The most commonly isolated NTM species were *M. abscessus* (37%) and *M. simiae* (37%). A significantly higher risk of *M. abscessus* colonization was observed in patients under 15 years of age (*p* = 0.039). Among those with positive cultures, 55.6% met the 2007 ATS microbiological criteria, and the overall prevalence of NTM pulmonary disease was 15.9%. NTM isolation was significantly associated with increased colonization by *Aspergillus* spp. (*p* < 0.0001), while it was associated with a lower frequency of *S. aureus* colonization (*p* < 0.0001) and showed no significant association with *P. aeruginosa* prevalence (*p* = 0.2991) [66].

Eltringham I. et al. conducted a study aiming to compare the efficacy of RGM medium with the conventional mycobacterial growth indicator tube (MGIT) for the isolation of rapidly growing mycobacteria from sputum samples of CF population. The study included 187 CF patients, 7–56 years old, from King’s College Hospital in London, and reported an overall prevalence of 15%. The study concluded that the RGM medium exhibits comparable sensitivity to MGIT and can be easily implemented in routine laboratory practice [67].

The prospective study by Preece CL et al., involving 210 patients from three hospitals in the United Kingdom, aimed to evaluate the efficacy of a novel selective culture medium (RGM medium) for the isolation of rapidly growing NTM from sputum samples of CF patients, in comparison to the established *Burkholderia cepacia* selective agar (BCSA). The authors reported an NTM infection prevalence of 15.7% and a *MABSC* infection prevalence of 10%. The RGM medium demonstrated significantly superior sensitivity (98%) compared to BCSA (31%) for rapidly growing NTM [68].

Salsgiver EL et al. conducted a retrospective study in the United States using data from the CFFPR, aiming to calculate the annual trends of incidence and prevalence of selected CF pathogens. In 2012, the overall prevalence of NTM infections among CF patients aged ≥12 years was 12.0%. Prevalence of *MAC* was 6.94%, 5.03% for *MABSC*, and 0.82% for other NTM species. The annual incidence of NTM infection in 2012 was estimated at 5.52% for patients aged 11–17 years and 6.06% for those aged 18–25 years. Moreover, a statistically significant increasing trend in overall NTM prevalence was observed (+3.9% per year, *p* < 0.05), particularly for *MAC* (+7.4% per year, *p* < 0.05), whereas the incidence of *MABSC* remained stable throughout the 2010–2012 period [69].

According to Viviani L. et al. the overall prevalence of NTM infections was reported at 2.7% in a cross-sectional European study based on data from 13,593 pediatric and adult patients recorded in the European Cystic Fibrosis Society Patient Registry (ECFSPR). Patients with a positive NTM culture were, on average, older and had significantly lower FEV1 values compared to NTM-negative patients (*p* < 0.0001). Furthermore, the possibility of NTM isolation increased by 17.5% for every 10-year increase in age and decreased by 7.5% for every 10-percentage-point increase in predicted FEV1. Colonization with *S. maltophilia* and *P. aeruginosa* was more frequent among NTM-positive patients, with *S. maltophilia* being associated with more than double the odds of NTM presence (*p* < 0.0001). Additionally, the presence of allergic bronchopulmonary aspergillosis (ABPA) was significantly associated with NTM infection, increasing the odds by 2.36-fold (*p* < 0.0001) [70].

The retrospective case–control study by Cavalli Z. et al., conducted in the CF Center of Lyon, included 401 pediatric and adult patients and reported an overall prevalence of NTM infection of 12% over the six-year period. Although the authors noted an increasing trend in NTM cases over time, specific annual incidence rates were not provided. *MAC* was isolated in 56.3% of cases, while *MABSC* accounted for 37.5%. The highest frequency of NTM isolation occurred among adolescents aged 13–17 years. *MABSC* was predominantly recovered in children (67%), with a mean age of 17.1 years, whereas *MAC* was equally distributed between children and adults, with a mean age of 20.2 years. NTM pulmonary disease was diagnosed in 31.3% of the NTM-positive cases. Although infections due to *MAC* were associated with a higher proportion of NTM-PD compared to *MABSC* (37.3% vs. 27.8%), this difference was not statistically significant (*p* = 0.74). Notably, colonization with *S. aureus* was associated with a fourfold increased risk of NTM isolation (*p* = 0.007), and a significant association was also observed between NTM infection and allergic bronchopulmonary aspergillosis (ABPA) (*p* = 0.011). Regarding pulmonary function, the mean annual decline in FEV_1_ was −1.68% in NTM-positive patients versus −0.58% in controls (*p* = 0.047), indicating a more rapid deterioration of lung function in those with NTM infection [71].

Plongla R. et al. aimed to compare the effectiveness of the RGM medium with other culturing methods for the isolation of NTM from samples of CF patients, as well as to evaluate the utility of MALDI-TOF MS for species identification. In this prospective study, respiratory specimens from adult and pediatric CF patients were collected at UNC Health Care (USA) as part of routine culture for common pathogens and mycobacteria. The overall prevalence of NTM infection was estimated at 14.2%. The combined use of culture methods identified the *MABSC* as the most frequently isolated species (prevalence 5.5%), followed by the *MAC* (prevalence 3.3%), with comparable recovery rates between the RGM and AFBC methods [72].

In the retrospective study conducted by Aiello TB et al., which included 117 patients followed at a cystic fibrosis reference center in Brazil, the overall prevalence of NTM infection was 6%. The most commonly isolated species were *M. abscessus* and *M. avium*. Most patients were already co-infected with *P. aeruginosa*, *S. aureus*, while *B. cepacia* and *Aspergillus* spp. were observed less commonly. The mean FEV1 among patients with NTM infection was notably low, at 40% [73].

In the study by Scohy A. et al., which was conducted at the Saint-Luc University Hospital in Brussels, the performance of the RGM medium for isolating NTM from sputum samples from CF patients was evaluated. A total of 124 patients were prospectively assessed, and the recorded prevalence of NTM infection was 10.5%. The majority of isolates was *MABSC* (55%). The study demonstrated that the RGM medium is a reliable, simple, and effective alternative for NTM culture in CF patients [74].

Eikani MS et al. conducted a retrospective case–control study in Milwaukee, USA, reporting a prevalence of NTM infection of 8.3%. However, they noted that this figure might be underestimated due to the irregular screening practices for NTM in patients with CF. The most commonly isolated species was *MAC*, followed by *MABSC*, with no significant difference in mean age at infection between the two. A significant association was found between age of CF diagnosis and infection with rapidly growing mycobacteria (RGM). Patients infected with slowly growing NTM had a significantly younger age of CF diagnosis (median 1.2 months [range: 0.1–2.8 months]) compared to those infected with rapidly growing NTM (4.5 months [range: 1.5–84.3 months]) and NTM-negative individuals (2.4 months [range: <1–164 months]). The difference in age of CF diagnosis was statistically significant between patients infected with rapidly growing NTM and those without NTM infection (*p* = 0.013). Early diagnosis through newborn screening and access to modern therapies contribute to the prevention and better management of NTM infections. Delayed diagnosis is associated with poorer health status, malnutrition, and advanced lung disease, all of which may increase the risk for infection with RGM. Although FEV_1_ did not differ significantly before and after NTM infection, the FEF25–75 index showed a significant decline in NTM-positive groups, indicating worsening small airway function. The greatest decline in FEF25–75 was observed in patients with more than four positive cultures for RGM, supporting an association with more severe and acute clinical manifestations. *P. aeruginosa* and *S. aureus* were the most commonly co-isolated pathogens in NTM-positive patients. However, no statistically significant association was found between a history of ABPA and NTM infection in this analysis [75].

In the retrospective study conducted by Adjemian J. et al., using data from the Cystic Fibrosis Foundation Patient Registry (CFFPR), the overall prevalence of NTM infection was estimated at 20%, with a significant age-related increase (19% in patients aged 12–17 years, 20% in those aged 18–59 years, and 29% in individuals over 60 years; *p* = 0.0001). Among patients with NTM, *MAC* was isolated in 61% (prevalence 12%), *M. abscessus* in 39% (prevalence 8%), and other NTM species in 21% (prevalence 4%). In patients over 60 years of age, *MAC* prevalence reached 20%, while *M. abscessus* prevalence remained stable at 10%. Patients infected with either *MAC* or *M. abscessus* were significantly more likely to have higher FEV_1_% predicted (*p* < 0.01). *MAC*-infected individuals were older on average (28.36 ± 12.8 years), while *M. abscessus*-infected patients were younger (26.2 ± 11.9 years) compared to those without NTM infection (27.06 ± 11.6 years; *p* < 0.0001). The annual prevalence of NTM infection increased from 11.0% in 2010 to 13.4% in 2014 (*p* = 0.0008). A significant rise in annual prevalence was also observed among patients with a median age of 40 years—from 10.9% in 2010 to 13.3% in 2014 (*p* = 0.0003)—but not in those over 40 years (*p* = 0.07). The prevalence of *MAC* infection increased from 5.8% in 2010 to 6.9% in 2014, whereas the prevalence of *M. abscessus* remained stable at 5.1% across all years (*p* = 0.9). In the five-year regional prevalence analysis, *M. abscessus* predominated in Hawaii (50%), Florida (17%), and Louisiana (16%), while *MAC* was most common in Nevada (24%), Kansas (21%), and both Hawaii and Arizona (20% each). Co-isolation of other pathogens such as *Aspergillus* spp. and *S. maltophilia* was associated with an increased risk of NTM infection. *M. abscessus* infections were more commonly linked to southern U.S. states and were less frequent in female patients, whereas *MAC* infections were associated with an increased risk of co-infection with *Staphylococcus aureus* [76].

A large retrospective study by Hatziagorou E. et al., using data from 42,958 patients included in the European Cystic Fibrosis Society Patient Registry (ECFSPR) over the period 2011–2016, reported overall prevalence of NTM infection of 3.3% and incidence of 1.4%. The prevalence of NTM showed a rising trend across all age groups, with higher rates observed in middle- and high-income countries. In contrast, differences in incidence rates across countries were relatively small [77].

In a retrospective cohort study conducted in the U.S.A., Low D. et al. analyzed data from 17,177 patients aged over 10 years, aiming to evaluate screening practices for NTM and to identify clinical factors influencing the timing of NTM testing. The authors reported that the proportion of patients with any positive NTM culture increased from 10.9% in 2010 to 12.9% in 2014. Among the positive NTM cultures in 2014, *MAC* was the most frequently isolated species (51.2%), followed by *M. abscessus* (37.4%). The study showed that although screening practices increased over time, testing decisions were primarily based on clinical criteria (such as age, lung function, and infections), which did not always correlate with positive NTM cultures. Annual routine screening appeared to be more effective, as centers implementing this strategy identified a higher number of positive cases [78].

Ho D. et al. analyzed retrospectively medical records of 183 CF patients of all ages treated at university hospitals on Réunion Island, a French overseas region in the Indian Ocean. The overall prevalence of NTM infection was 26.4%, rising to 36.9% among patients older than 12 years. The incidence remained stable over the 13-year period, at approximately 3% per year. *M. abscessus* (60.8%) and *M. avium* (27.4%) were the most frequently isolated species, while *M. simiae* was identified in 7.8% of cases. Co-infection with *A. fumigatus* was observed in 39.2% of patients. According to ATS criteria, 56.9% of patients met the definition for NTM infection. In multivariate analysis, patients who fulfilled the ATS criteria were significantly more likely to be infected with *M. abscessus* (*p* = 0.026) [79].

The retrospective study by Ademhan D. et al. in Turkey included 485 CF patients, both children and adults, and reported a NTM infection incidence of 2.1%. The most frequently isolated species was the *MABSC* (80%), while *M. avium* and *M. szulgai* were detected less frequently. The mean age of patients with NTM infection was 19 years. Among those with active infection, FEV1 decreased by 17% compared to the previous year. All patients were co-infected with other pathogens, most commonly *S. aureus* and *P. aeruginosa* [80].

The study by Foote SL. et al., using data from 979 CF patients over the age of 12 from the Cystic Fibrosis Foundation Patient Registry (CFFPR), defined cases as individuals residing in Florida for at least two consecutive years who developed a new positive NTM culture following at least one negative culture. The incidence of NTM infection recorded in this population was 26.7%. The most frequently isolated species were *MAC* (41.8%) and *M. abscessus* (45.2%) [81].

The case–control study by Lipner EM et al. was conducted in Colorado, USA, with the aim of identifying water components associated with the risk of NTM infection in CF patients. Cases were defined as individuals who had at least one NTM-positive culture while residing in Colorado at the time of initial diagnosis. A total of 193 patients with positive NTM cultures participated, of whom 76.2% were infected with *MAC* (*Mycobacterium avium*, *M. intracellulare*, or *M. chimaera*) and 42.3% with *M. abscessus* (*M. abscessus*/*chelonae*, *M. massiliense*, or *M. bolletii*). The study found that the presence of molybdenum in environmental water sources (e.g., rivers and lakes) was associated with an increased likelihood of NTM infection in individuals with CF, with a stronger association observed specifically for infections caused by *M. abscessus* [82].

Zomer D. et al. conducted a retrospective case–control study including 1516 pediatric and adult patients from the Dutch CF Registry and observed an increase in the annual prevalence of NTM infections, from 1% in 2013 to 3.6% in 2019. *M. abscessus* (47.1%) and *MAC* (30.9%) were the most common species, with slightly lower prevalence in the pediatric population. Treatment was more frequently administered to individuals infected with *M. abscessus* (51.6%) compared to those with *MAC* (26.8%) or other NTM species (16.0%). Among children with NTM infection, significantly higher rates of co-infection with *S. maltophilia* and *A. fumigatus* were observed (*p* < 0.001), along with an increased frequency of ABPA two years after isolation (*p* = 0.007). FEV_1_ was already lower one year prior to isolation (81.5% vs. 88.6%, *p* = 0.024), showed >10% decline the following year (*p* = 0.002), and remained significantly lower two years later (*p* = 0.01). In contrast, among adults with NTM, no significant differences were found in lung function, co-infections, or ABPA incidence compared to the control group [83].

Wetzstein N. et al. conducted a retrospective study using data from 6295 patients recorded in the German Cystic Fibrosis Registry. During the period 2015–2020, the prevalence of NTM infections ranged from 1.9% to 3.0%. Specifically, positive NTM cultures were reported in 1.9% of patients in 2015, 2.4% in 2016, 3.0% in 2017, 2.9% in both 2018 and 2019, and 2.8% in 2020. The most frequently isolated species was *M. abscessus* (46.6–62.3% of cases), followed by *MAC* (15.1–36.3%). Other NTM species, such as *M. kansasii*, *M. fortuitum*, *M. gordonae*, and *M. chelonae*, made up 19.6% to 23.7% of isolates. Among children, *M. abscessus* was most commonly isolated (45.9% to 78.1%), while *MAC* was identified in 9.4% to 36.1% of cases. In adults, *M. abscessus* remained the predominant species (40.3% to 56.1%), although other NTMs, including *MAC*, showed increasing relevance (17.6% to 36.4%) [84].

In a retrospective cohort study, Ricotta EE. et al. analyzed data from patients over the age of 12 in the US Cystic Fibrosis Foundation Patient Registry (CFFPR) from 2011 to 2018 and estimated the prevalence of NTM infection at 23%. NTM isolation was recorded in 10% of patients receiving CFTR modulator therapy, compared to 33% among those not receiving such treatment. Furthermore, for each additional year of CFTR modulator use, the odds of positive NTM culture decreased by 37% (OR: 0.60, 95% CI: 0.58–0.62). A significant delay in the onset of NTM isolation was also observed in the treatment group, with a mean difference of 2.1 years. These findings suggest that CFTR modulator therapy is associated with a reduced risk of NTM isolation [85].

In the study by Mercaldo RA et al., which included data from 25,305 CF patients over the age of 12 from the Cystic Fibrosis Foundation Patient Registry (CFFPR) during the period 2010–2019, the recorded incidence of NTM infection was 14.3%. Focusing on detecting high-risk geographic regions, the authors applied five different statistical methods and identified 25 counties across five states in the U.S. as areas of concern. Consistently high rates were observed in regions of southern Florida, New York, and Kansas City, regardless of the method used. The identification of such areas suggests a potential influence of environmental factors and highlights the need for public health interventions and environmental surveillance in vulnerable geographic regions [86].

Marshall JE et al. used data from the Cystic Fibrosis Foundation Patient Registry (CFFPR) in a retrospective study to estimate the annual incidence of NTM infection among CF patients in the United States. The average annual incidence was 58 cases per 1000 individuals. The northeastern region exhibited the highest prevalence of *MAC*, while the southern region showed the highest prevalence of *M. abscessus*. From 2010 to 2019, the annual incidence of NTM infection in the U.S. increased significantly by 3.5% per year [87].

In the retrospective study by Steindor M. et al., who analyzed the data from 6295 patients in the German Cystic Fibrosis Registry from 2016 to 2020, the annual prevalence and incidence of NTM infections were found to remain relatively stable, ranging between 7.53–8.76% and 3.31–4.95%, respectively. *MABC* was the most frequently isolated NTM species throughout the study period, with relatively stable prevalence (3.63–4.47%) and incidence rates (1.25–1.93%). In contrast, a mild increase in *MAC* prevalence was observed, from 2.15% in 2016 to 3.08% in 2020, although its incidence fluctuated. In this study, NTM detection was significantly associated with age (per 10 years: OR = 1.24, 95% CI: 1.07–1.43, *p* < 0.05 for prevalence; OR = 1.18, 95% CI: 1.00–1.38 for incidence), low FEV1 (<40% vs. 40–70%: OR = 1.46 and OR = 1.77, respectively, *p* < 0.05), and the presence of ABPA (OR = 1.70, 95% CI: 1.02–2.83). Detection of *A. fumigatus* was also associated with increased NTM prevalence (OR = 1.63, *p* < 0.05). On the contrary, chronic infection with *P. aeruginosa* was linked to a reduced likelihood of NTM detection. Comparing *MAC* and *MABC* cases, *MAC* patients were older (median age 30 vs. 23 years, *p* < 0.0001) and had lower FEV1 at the time of first isolation (*p* = 0.0417). The effect of CFTR modulator therapy on NTM detection remains unclear. While previous U.S. data (2011–2018) suggested a modest reduction in NTM risk among patients receiving CFTR modulators, this association was not confirmed in the current study. Nevertheless, the presence of the delF508 genotype, which qualifies patients for these therapies (along with other rare mutations), was associated with a lower risk of NTM infection. The authors note that further investigation is needed, especially as more effective modulators become available for younger CF patients [88].

## 4. Discussion

A total of 78 studies met the inclusion criteria of this systematic review. The studies covered time periods ranging from 1990 to 2024. In terms of geographic distribution, they originated from Europe, North America, Latin America, the Middle East, and Australia, with the United States, the United Kingdom, and France being the most frequently represented countries. Most publications came from Europe or North America. The majority of studies involved mixed populations of pediatric and adult patients, while 13 studies focused exclusively on pediatric cohorts.

### 4.1. Prevalence of NTM Infections

Among studies focused exclusively on pediatric populations, overall prevalence ranged from 0% [11] to 32.2% [34], while those involving only adult patients reported values between 1.7% [26] and 32.5% [25]. In mixed-population studies, Seddon P. et al., 2009 [55] reported a prevalence of 3.3% in children and 5% in adults, while Devine M. et al., 2004 [48] recorded 0.9% in children and 3.0% in adults. The highest prevalence rates were observed in Spain (Campos-Herrero et al. [66]) and the United Kingdom (Al-Momani H. et al., 2017 [12]), while multiple studies from the United States repeatedly reported high prevalence rates [13,18,23,25,28,34,76,85]. NTM infection was not limited to adults, as several studies included pediatric or mixed-age populations, highlighting the wide distribution across all age groups. Notably, most studies reporting high NTM prevalence were based on small sample sizes, which may overestimate prevalence due to reduced population representativeness. On the contrary, studies involving larger cohorts reported prevalence rates ranging from 1.9% [84] to 23% [85].

Annual prevalence was reported in several studies, ranging from 0.9% (Esther Jr. et al., 2005 [30], a retrospective observational study in children) to 14.5% (in 2011, Bar-On O. et al., 2015 [62], a retrospective observational study including both children and adults). Point prevalence was also reported in some studies. For example, Radhakrishnan DK. et al. (2009) conducted a prospective observational study in a pediatric Canadian cohort (n = 190) and reported a point prevalence of 6.1% for the year 2004 [31]. Likewise, Leitritz L. et al. (2004) conducted a prospective observational study in adults in Germany (n = 91), reporting a point prevalence of 3.3% for the year 1999 [16].

The meta-analysis by Reynaud et al. (2020) estimated that approximately 6% of patients with cystic fibrosis had at least one NTM positive culture, based on a large sample of 23,418 individuals from various geographic regions [89].

The recent systematic review and meta-analysis by Prieto et al. (2023) [90], which included 95 studies—primarily from Europe (42%) and North America (33%)—mostly examined mixed populations of pediatric and adult CF patients. The global annual prevalence of NTM infection in individuals with CF was estimated at 7.9% (95% CI: 5.1–12.0%), with substantial heterogeneity between studies (I^2^ = 99%). The authors found that studies with smaller sample sizes, as well as those conducted outside North America and Europe, showed significantly different prevalence. Specifically, studies from outside these regions reported, on average, a 1.5% lower prevalence. Moreover, studies with fewer than 1000 participants tended to report higher prevalence rates compared to those with over 3000 patients when all other factors were the same. Reported period prevalence rates ranged from 6.6% to 19%. Meta-analysis of period prevalence was not performed due to variability in the duration of included studies, which contributed to increased heterogeneity in prevalence estimates [90].

### 4.2. Incidence

The incidence of NTM infections was reported in a limited number of studies, with rates ranging from 0.9% to 26.7%, depending on the study population and the duration of follow-up. In the study by Bar-On O. et al. (2015) in Israel, the annual incidence of NTM infection increased from 0% in 2002 to 9% in 2011 [62]. Campos-Herrero MI et al. (2016) observed considerable fluctuations in annual incidence, reaching up to 14.3% in 2002, whereas no new NTM isolations were recorded in four of the studied years (2006, 2010, 2011, and 2012) [66]. Ho D. et al. (2021) reported a stable annual incidence of approximately 3% over a 13-year period (2002–2015) [79]. In the United States, Salsgiver EL. et al. (2016) estimated the annual incidence of NTM infection in 2012 at 5.52% among individuals aged 11–17 years and 6.06% among those aged 18–25 years [69]. In a more recent study by Marshall JE. et al. (2023), the mean annual incidence was reported as 58 cases per 1000 individuals, with a 3.5% annual increase in incidence between 2010 and 2019 in the U.S. [87]. Hatziagorou E. et al. (2019) recorded in Europe an incidence of 1.4% for the period 2011–2016 [77]. Similarly, in a retrospective study by Steindor M. et al. (2023), the incidence of NTM infections in Germany remained relatively stable, ranging from 3.31% to 4.95% [88]. Ahmed MI et al. (2019) reported incidence 14% over a five-year follow-up period [35]. In contrast, Foote SL. et al. (2021) documented a notably higher incidence of 26.7% [81].

In the systematic review by Prieto et al. (2023) [90], most studies reporting NTM infection incidence had small sample sizes (≤110), with annual incidence rates below 10%. The highest incidence rate (14.3%) was reported in a study with only 44 participants, whereas the lowest rates (1.3–1.8%) were observed in the study with the largest sample size, conducted between 2011 and 2016 [90].

### 4.3. NTM Species

The most frequently isolated NTM species were the *Mycobacterium abscessus* complex (*MABSC*) and the *Mycobacterium avium* complex (*MAC*). In some studies, *MABSC* accounted for over 50% of positive cultures [20,28,33,39,51,67,73,74,79], whereas other studies reported higher proportions of *MAC* [23,31,34,47,56,57,59,71,76,78,82]. Less commonly reported species included *M. simiae*, *M. gordonae*, *M. chelonae*, and *M. xenopi*. Studies examining the epidemiology of NTM species by age groups indicated that *MABSC* was more frequently isolated in younger patients, in contrast to *MAC*, which predominated in older age groups [46,47,50,53,54,71]. According to the recent systematic review by Prieto et. al, *Mycobacterium abscessus* complex (4.1%) and *Mycobacterium avium* complex (3.7%) were identified as the most common NTM species. The prevalence of *MAC* was significantly lower in Europe (1.7%) compared to North America (7.8%), whereas no regional differences were observed for *MABSC* [90].

### 4.4. Lung Function

Almost 30% of all studies reported data on FEV1. In five of them, no significant difference was found in FEV1 between NTM-positive and NTM-negative patients [14,31,36,44,62]. In the study by Eikani MS et al. (2018), FEV1 did not differ before and after NTM infection, but the FEF25–75 index was significantly lower in patients with positive cultures [75]. In studies by Kilby JM et al. (1992) and Sermet-Gaudelus I. et al. (2003), FEV1 values showed considerable variability [13,46]. Binder AM. et al. (2013) reported no difference in FEV_1_ among patients infected with MAC, but observed higher FEV1 values in those with *M. abscessus* [57].

Richter WJ et al. (2021), Olivier KN et al. (2002), and Adjemian J et al. (2018) noted higher FEV1 in NTM-positive patients, though no interpretation or explanation was provided by the authors [23,47,76]. Esther CR Jr et al. (2005) recorded rapid decline of FEV1 among patients with NTM-positive sputum cultures [30], while Cavalli Z. et al. (2017) reported an annual FEV1 decline rate of −1.68% [71]. Decline of FEV1 was also documented in studies by Levy I et al. (2008) [53], Viviani L et al. (2016) [70], Ademhan D et al. (2021) [80], Abidin NZ et al. (2022) [39], Steindor M et al. (2023) [88], Aiello TB et al. (2018) [73], Martiniano SL et al. (2014) [59], and Örlős Z et al. (2024) [27]. In the study by Zomer D et al. (2022), decline was observed only in the pediatric population [83].

Esther CR Jr et al. (2010) [56] also found an association between FEV1 and patient age. Patients with *M. abscessus* exhibited an additional annual decline in FEV1 compared to NTM-negative individuals. Overall, the group with chronic NTM infection experienced a greater yearly decline in predicted FEV1 (−2.33%) [56]. Hughes DA et al. (2021) reported that patients with *MAC* who received treatment had lower FEV1 values [38].

### 4.5. Nontuberculous Mycobacteria Pulmonary Disease (NTM-PD)

Fourteen studies reported the proportion of patients with NTM-positive sputum cultures who met the American Thoracic Society (ATS) diagnostic criteria for NTM pulmonary disease (NTM-PD). In the study by Paschoal IA et al. (2007), none of the patients fulfilled the diagnostic criteria for NTM-PD [19]. Reported proportions varied, reaching as high as 83% [58].

In seven of these studies, the majority of NTM-PD cases were associated with *Mycobacterium abscessus* complex (*MABSC*) [30,49,50,54,62,79,83], while in the study by Cavalli Z et al. (2017) [71], *Mycobacterium avium* complex (MAC) was the predominant species. The prevalence of NTM-PD was specifically reported in four studies [20,29,49,62], ranging from 1,9% (France, 1997) to 11.9% (Israel, 2009). In the study by Bar-On O et al. (2015) [62], the prevalence of NTM-PD appeared to decline from 11.9% in 2009 to 5.5% in 2011.

### 4.6. Co-Colonization with Other Microorganisms

Thirty studies investigated bacterial colonization in patients with NTM infection, revealing frequent co-infection with other respiratory pathogens. *Pseudomonas aeruginosa* was the most commonly identified microorganism, with reported rates ranging from 10% [47] to 95.2% [53]. *Staphylococcus aureus* appeared less frequently. The fungus *Aspergillus* spp. and allergic bronchopulmonary aspergillosis (ABPA) were also commonly reported. In several studies, *Stenotrophomonas maltophilia* was additionally detected.

According to the meta-analysis by Reynaud et al., co-infection with *Aspergillus fumigatus*, *Staphylococcus aureus*, or *Stenotrophomonas maltophilia* was strongly associated with an increased risk of NTM infection, possibly as a result of greater inflammation or more severe lung damage [89].

### 4.7. Geographic Distribution

There is significant variability in the geographic distribution of NTM infections. In Europe, the overall prevalence of NTM infection according to various studies ranged from 1.7% [26] to approximately 25% [17], with three notable exceptions. Two studies from Spain reported higher rates of 30.4% [22] and 40.9% [66], while the study by Al-Momani H. et al., 2017, focusing on gastrostomy fed CF patients in U.K., reported a prevalence of 43% [12]. Recent European studies have highlighted rising trends in the prevalence of NTM infections, with Hungarian data showing an increase from 4.7% in 2020 to 12.9% in 2022 [27], while a French study reported rates of 7.2%, 8.7%, and 10% in the three years prior to elexacaftor–tezacaftor–ivacaftor initiation, followed by a notable decrease to 1.7% one year after treatment onset [26]. In the United States, prevalence rates ranged from 3.9% [30] in 2005 to 32.5% [25] in 2022, with the most recent study reporting a prevalence of 24.1% in 2024 [28].

Some studies highlighted regional variation within countries. For example, in Israel, higher prevalence was observed in central and southern regions compared to the north [53], while in the U. K., southeastern England exhibited rates up to 7.5%, in contrast to 1.9% in Northern Ireland [55]. In the U.S., consistently high NTM prevalence was reported in regions such as south Florida, New York, and Kansas City [86]. Additionally, the study by Kopp BT. et al., 2015 [65] revealed higher rates of at least one positive culture in the western and southern regions of the country. Bouso JM et al., 2017 found that patients living within 500 m of a water source had a 9.4-fold increased likelihood of NTM isolation [34].

Geographic patterns were also observed regarding species distribution. *Mycobacterium abscessus* was more frequently identified in regions such as Hawaii, Florida, and Louisiana, whereas *Mycobacterium avium* complex predominated in Nevada, Kansas, and Arizona in U.S. studies [76]. Similarly, in the Paris region of France, *MABSC* was more common than *MAC* [54]. In contrast, no differences in species distribution were observed between urban and rural areas in the Netherlands [83]. These findings support the hypothesis that environmental and climatic factors may influence the global distribution of NTM species.

### 4.8. CFTR Modulators

CFTR modulator therapy has introduced a new era in the treatment of cystic fibrosis. However, their impact on the natural course of nontuberculous mycobacterial infections remains unclear, as comparative studies evaluating the incidence, prevalence, or outcomes of NTM infections before and after the initiation of CFTR modulator therapy are currently lacking. The widespread use of CFTR modulators has been associated with reduced sputum production and a lower risk of NTM infection among individuals with CF. Despite these promising findings, CF patients remain a high-risk population, and continued surveillance is essential to monitor disease burden and epidemiological trends [87].

In the retrospective cohort study by Ricotta EE et al. (2022) [85], NTM isolation was reported in a lower proportion of patients receiving CFTR modulators (10%) compared to those not receiving such therapy (33%). Consequently, CFTR modulator treatment was considered to be associated with a reduced risk of NTM isolation [85]. Despite these findings from the U.S., data from the study by Steindor M. et al. (2023) [88] in Germany did not confirm this association, and the impact of CFTR modulators on NTM detection remained uncertain. The authors highlighted the need for further investigation, particularly as modulators are developed for use in younger age groups [88]. Mianowski L. et al. (2024) [26], in France, reported rates as high as 10% before CFTR modulator initiation and a significant reduction to 1.7% one year after treatment onset. The observed reduction in lung bacterial colonization in this cohort seemed to have begun even before the introduction of ETI. According to the authors, this trend may be partially explained by the prior use of ivacaftor/lumacaftor in many patients, as well as by decreased routine clinic visits and sputum sampling during the COVID-19 pandemic. Reduced patient attendance, fewer collected sputum samples, and pandemic-related behavioral changes, including lockdown measures, likely contributed to lower detectable bacterial prevalence. This highlights that the absence of pathogen documentation does not necessarily reflect true eradication. These findings emphasize the need to interpret prevalence data cautiously and suggest that future guidelines for sputum monitoring and antibiotic strategies should consider such external factors [26].

### 4.9. Strengths and Limitations

The differences of study findings could not be easily interpreted as there are centers with higher screening intensity and the lab methodology differed. For this reason, further multicenter studies are needed following the same screening and lab protocol.

Strengths of this review include the broad coverage of both pediatric and adult populations across diverse regions. Moreover, the integration of clinical perspectives—such as lung function decline, co-infections, and the potential role of CFTR modulators—enhances its relevance for clinical practice.

A limitation of this review is that the literature search was restricted to PubMed. We acknowledge that additional databases (e.g., Embase, Scopus, Web of Science, Cochrane Library, and regional databases) may have yielded further studies. However, resource constraints precluded a full multi-database search. Furthermore, another limitation is the lack of risk of bias assessment of the included studies. A formal risk of bias assessment was not performed because many of the included studies did not provide sufficient methodological details to allow consistent and reliable evaluation across different study designs. Applying existing risk of bias tools in this context could have resulted in incomplete or potentially misleading results. Instead, we have noted in the text the main weaknesses of the included studies, including small numbers of participants, retrospective designs, and variations in outcome definitions. These factors should be taken into account when interpreting the certainty of the available evidence.

## 5. Conclusions

The findings of this systematic review suggest

- An increasing trend in the prevalence of nontuberculous mycobacteria infection among patients with cystic fibrosis, as reported in multiple recent studies. This highlighted rise may partially reflect improved surveillance and diagnostic awareness over time.
- Impaired lung function and microbial co-colonization appear to be significant risk factors for NTM infection.
- The introduction and widespread use of CFTR modulators may contribute to a decline in NTM infections, although current evidence remains insufficient to draw firm conclusions.
- Further multicenter prospective studies are needed in order to
  ○ Clarify the possible effect of CFTR modulators on NTM prevalence in CF.
  ○ Specify the other factors that affect the epidemiology of NTM prevalence in CF.

## Figures and Tables

**Table 1:** Characteristics of the included adult studies.

	Study	Year	Study Population	Study Design	Country	Prevalence (%, n/N)	MAC/ MABSC (%, n/N)
1	Kilby JM et al., 1992 [13]	1981–1990	87	Retrospective observational	U.S.A.	Overall: 20.0 (17/87)	-
2	Aitken ML et al., 1993 [14]	1990–1991	64	Prospective cross-sectional	U.S.A.	Overall: 12.5 (8/64)	-
3	Bange FC et al., 2001 [15]	1997–1999	214	Retrospective	Germany	Overall: 7.0 (15/214)	-
4	Leitritz L. et al., 2004 [16]	1999–2001	91	Prospective observational	Germany	Overall: 11.0 (10/91) Point: 3.3 (-)	-
5	Girón RM et al., 2005 [17]	1997–2001	28	Prospective cohort	Spain	Overall: 25 (7/28)	-
6	Chalermskulrat W. et al., 2006 [18]	1990–2003	146	Retrospective cohort	U.S.A.	Prior transplantation: 19.7 (26/132) Post-transplantation: 13.7 (20/146)	45 (13/29 isolates) /41 (12/29 isolates)
7	Paschoal IA. et al., 2007 [19]	2003–2004	54	Retrospective cross-sectional	Brazil	Overall: 11.0 (9/54)	-
8	Coolen N. et al., 2015 [20]	2006–2010	347	Case–control	France	Overall: 12.4 (41/347)	48.7 (20/41) /58.5 (24/41)
9	Al-Momani H. et al., 2017 [12]	2017	16	Observational cross-sectional	United Kingdom	Overall: 43 (7/16)	-
10	Sherrard LJ. et al., 2017 [21]	2001–2013	434	Retrospective observational cohort	Australia	Overall: 14 (54/375)	-
11	Fernández-Caso B.et al., 2019 [22]		92	Retrospective observational	Spain	Overall: 30.4 (28/92)	-
12	Richter WJ. et al., 2021 [23]	2007–2018	254	Retrospective cohort	U.S.A.	Overall: 19.7 (50/254)	58 (29/50) /36 (18/50)
13	Wyrostkiewicz D. et al., 2022 [24]	2010–2020	151	Retrospective observational	Poland	Overall: 7 (11/151)	55 (6/11)/9 (1/11)
14	Gross JE. et al., 2022 [25]	2012–2018	507	Retrospective cohort	U.S.A.	Overall: 32.5 (165/507)	-
15	Mianowski L. et al., 2024 [26]	2020–2021	198	Retrospective observational	France	2Y prior ETI: 7.2 (14/194) 1Y after ETI: 1.7 (3/179)	-
16	Örlős Z. et al., 2024 [27]	2020–2022	232	Retrospective cohort	Hungary	Point: 16.8 (39/232) Annual: 5.7 (11/192) (2020) –12.9 (30/232) (2022)	41 (16/39)/38.5 (15/39)
17	Gross JE et al., 2024 [28]	2013–2018	294	Retrospective observational	U.S.A.	Overall: 24.1 (71/294)	-/70.4 (50/71)

**Table 2:** Characteristics of the included pediatric studies.

	Study	Year	Study Population	Study Design	Country	Prevalence (%, n/N)	MAC/ MABSC (%)
1	Fauroux B. et al., 1997 [29]	1997	106	Prospective	France	Overall: 6.6 (7/106)	-
2	Esther Jr. et al., 2005 [30]	1993–2002	545	Retrospective observational	U.S.A.	Overall: BAL cohort: 6.1 (7/114) Registry cohort: 3.9 (17/431) Annual: 2.0 (-)	-
3	Radhakrishnan DK.et al., 2009 [31]	2004	98	Prospective cohort	Canada	Point: 6.1 (6/98)	66 (4/6)/33 (2/6)
4	Cândido PH et al., 2014 [32]	2009–2012	129	Prospective cohort	Brazil	Overall: 7.75 (10/129)	-
5	Satana D. et al., 2014 [33]	2003–2008	130	Retrospective observational	Turkey	Overall: 3.07 (4/130)	-/60.9 (14/23 isolates)
6	Bouso JM et al., 2017 [34]	2012–2015	65	Retrospective observational	U.S.A.	Overall: 32.2 (21/65)	66.7(14/21)/23.8 (5/21)
7	Ahmed MI et al., 2019 [35]	2012–2016	42	Prospective observational cohort	United Kingdom	-	-
8	Gardner AI. et al., 2019 [36]	2010–2015	5333	Retrospective cohort	United Kingdom	Overall: 5.4 (288/5333) Annual: 1.3–3.8 (-)	-
9	Yan J. et al., 2020 [37]	2013–2017	99	Retrospective	Australia	Overall: 11 (99/328 total population)	-/6.7 (22/328 total population)
10	Arvind B. et al., 2020 [11]	2013–2015	104	Prospective observational	India	0 (0/104)	-
11	Hughes DA et al., 2021 [38]	2011–2018	567	Retrospective cohort	United Kingdom	Overall: 10.4 (59/657)	-
12	Abidin NZ. et al., 2022 [39]	2016–2018	4687	Retrospective observational	United Kingdom	Overall: 6.5 (303/4687) Annual: 3.1–3.6 (-)	30.4 (92/303) /58.1 (176/303)
13	Singh J. et al., 2024 [40]	2002–2019	419	Retrospective observational cohort	Australia	Overall: 0.72 (-/419)	-

**Table 3:** Characteristics of included mixed-age studies.

	Study	Year	Study Population	Study Design	Country	Prevalence (%, n/N)	MAC/ MABSC (%)
1	Mulherin D. et al., 1990 [41]	1989	43	Prospective observational	United Kingdom	Overall: 2.3 (1/43)	-
2	Hjelte L. et al., 1990 [42]	1990	54	Prospective observational	Sweden	Overall: 9.3 (5/54)	-
3	Hjelt K. et al., 1994 [43]	1987–1988	185	Prospective cross-sectional	Denmark	Point: 1.6 (3/185)	-
4	Torrens JK et al., 1998 [44]	1989–1994	372	Retrospective case–control	United Kingdom	Overall: 3.8 (14/372)	-
5	Oliver A. et al., 2001 [45]	2000	37	Prospective observational	Spain	Overall: 16.1 (6/37)	-
6	Sermet-Gaudelus I. et al., 2003 [46]	1996–1999	296	Prospective cohort	France	Overall: 9.8 (29/296)	
7	Olivier KN. et al., 2003 [47]	2002	986	Prospective cross-sectional	U.S.A.	Overall: 13 (128/986)	72 (92/138)/16 (18/128)
8	Devine M. et al., 2004 [48]	2004	182	Retrospective cross-sectional	United Kingdom	Overall: Pediatric 0.9 (1/116) Adult 3.0 (2/66)	
9	Mussaffi H. et al., 2005 [49]	1997–2003	139	Retrospective cohort	Israel	Overall: 8.6 (12/139)	-
10	Pierre-Audigier C. et al., 2005 [50]	2000	386	Prospective cross-sectional	France	Overall: 8.1 (31/385)	21.2 (7/33)/39.4 (13/33)
11	Ferroni A. et al., 2006 [51]	2004–2005	289	Comparative	France	Overall: 11.0 (32/289)	5 (3/60 isolates) /72 (43/60 isolates)
12	Valenza G. et al., 2008 [52]	2006	60	Cross-sectional	Germany	Point: 13.3 (8/60)	-
13	Levy Ι. et al., 2008 [53]	2001–2003	186	Retrospective cross-sectional	Israel	Overall: 22.6 (42/186)	14.3 (6/42)/31 (13/42)
14	Roux AL. et al., 2009 [54]	2004	1582	Prospective cross-sectional	France	Overall: 6.6 (104/1582)	22 (23/104)/48 (50/104)
15	Seddon P. et al., 2009 [55]	2009	7122	Cross-sectional	United Kingdom	Overall: 4.2 (300/7122) Pediatric 3.3(110/3317) Adult 5.0 (190/3805)	-
16	Esther CR Jr et al., 2010 [56]	2000–2007	1216	Retrospective observational	U.S.A.	Overall:13.7 (166/1216) Mean annual: 10.8 (-)	59 (98/166)/41 (68/166)
17	Binder AM. et al., 2013 [57]	2003–2011	5403	Nested case–control	U.S.A.	Point: 4 (191/5403)	64 (122/191)/36 (69/191)
18	Qvist T et al., 2014 [58]	2012–2013	198	Prospective cohort	Denmark	Overall: 12 (23/198)	-
19	Martiniano SL. et al., 2014 [59]	2000–2010	650	Retrospective cohort	U.S.A.	Overall: 14.8 (96/650) Pediatric 9.6 (48/499) Adult 31.7 (48/151)	Pediatric 75 (36/48)/21 (10/48) Adult 69 (33/48)/27 (13/48)
20	Adjemian J. et al., 2014 [60]	2010–2011	10,527	Retrospective observational	U.S.A.	Overall: 14 (1384/10,527)	-
21	Raidt L. et al., 2015 [61]	2001–2011	94	Retrospective observational	Germany	Annual: 0 (0/94) 2001 –7.4 (7/94) 2011	-
22	Bar-On O. et al., 2015 [62]	2002–2011	110	Retrospective observational	Israel	Annual: 5 (4/79) 2003 –14.5 (16/110) 2011	24(-)/46(-) of all isolates
23	Phelippeau M. et al., 2015 [63]	2010–2014	354	Prospective observational	France	Overall: 7.1 (25/354)	32 (8/25)/48 (12/25)
24	Qvist T. et al., 2015 [64]	2000–2012	1411	Retrospective	Scandinavia	Overall: 11 (157/1411)	32 (51/157)/45 (70/157)
25	Kopp BT et al., 2015 [65]	2007–2012	30,896	Retrospective	U.S.A.	Overall: 8.1 (2512/30,896)	-
26	Campos-Herrero MI. et al., 2016 [66]	2002–2012	44	Retrospective observational	Spain	Overall: 40.9 (18/44) Mean annual: 14.1 (-)	-/37 (7/18)
27	Eltringham I. et al., 2016 [67]	2015	187	Prospective	United Kingdom	Overall: 15 (28/187)	-/90 (21/28)
28	Preece CL. et al., 2016 [68]	2014	210	Prospective observational	United Kingdom	Overall: 15.7 (33/210)	-/10 (21/210)
29	Salsgiver EL. et al., 2016 [69]	2010–2012	13,169	Retrospective observational	U.S.A.	Annual: 12(-)	6.94/5.03 of all pts tested
30	Viviani L. et al., 2016 [70]	2009	13,593	Cross-sectional	Europe	Overall: 2.7 (374/13,593)	-
31	Cavalli Z. et al., 2017 [71]	2009–2014	401	Retrospective case–control	France	Overall: 12 (48/401)	56.3 (27/48) /37.5 (18/48)
32	Plongla R. et al., 2017 [72]	2015–2016	487	Prospective	U.S.A.	Overall: 14.2 (69/487)	3.3 (-)/5.5 (-) of all isolates
33	Aiello TB. et al., 2018 [73]	2018	117	Cross-sectional observational	Brazil	Overall: 6 (7/117)	42.9 (3/7)/57.1 (4/7)
34	Scohy A. et al., 2018 [74]	2016–2017	124	Prospective	Belgium	Overall: 10.5 (13/124)	-/55 (11/20 isolates)
35	Eikani MS et al., 2018 [75]	2003–2023	360	Retrospective, case–control	U.S.A.	Overall: 8.3 (30/360)	-
36	Adjemian J. et al., 2018 [76]	2010–2014	16,153	Retrospective cohort	U.S.A.	Overall: 20 (3211/16,153) Annual: 11–13.4 (-)	61 (1949/3211) /39 (1249/3211)
37	Hatziagorou E. et al., 2019 [77]	2011–2016	42,958	Observational	Europe	Overall: 3.3 (-)	-
38	Low D. et al., 2020 [78]	2010–2014	17,177	Retrospective cohort	U.S.A.	Annual: 10.9 (890/8172) 2010 –12.9 (1462/11,348) 2014	51.2 (749/1.462) /37.4 (547/1.462)
39	Ho D. et al., 2021 [79]	2002–2015	171	Retrospective observational	Reunion Island	Overall:26.4 (51/171)	27.4 (14/51) /60.8 (31/51)
40	Ademhan D. et al., 2021 [80]	2012–2020	485	Retrospective cohort	Turkey	-	-
41	Foote SL. et al., 2021 [81]	2010–2017	979	Nested case–control	U.S.A.	-	-
42	Lipner EM et al., 2022 [82]	2007–2019	193	Nested case–control	U.S.A.	-	76.2 (147/193) /42.3 (82/193)
43	Zomer D. et al., 2022 [83]	2013–2019	1516	Retrospective case–control	Netherlands	Annual: 1 (14/1353) 2013 –3.6 (55/1516) 2019	30.9 (42/136 isolates)/47.1 (64/136 isolates)
44	Wetzstein N. et al., 2022 [84]	2015–2020	6295	Retrospective observational	Germany	Overall: 1.9 (106/5462 in registry) (2015) –3.0 (177/5869) (2017)	15.1 (16/106) 2015 –36.3 (65/179) 2020/46.6 (82/176) 2018 –62.3 (66/106) 2015
45	Ricotta EE. et al., 2022 [85]	2011–2018	17,403	Retrospective cohort	U.S.A.	Overall: 23 (4002/17,403)	-
46	Mercaldo RA. et al., 2022 [86]	2010–2019	25,305	Retrospective	U.S.A.	-	-
47	Marshall JE. et al., 2023 [87]	2010–2019	3771	Retrospective cohort	U.S.A.	-	-
48	Steindor M. et al., 2023 [88]	2016–2020	6295	Retrospective observational	Germany	Annual: 7.53 (133/1767 tested) 2016 –8.76(185/2112 tested) 2020	2.15 (38/1767 tested) 2016 –3.08 (65/2112) 2020/ 3.63 (84/2317) 2019 –4.47 (79/1767) 2016

## Abbreviations

ABPA	Allergic Bronchopulmonary Aspergillosis
ATS	American Thoracic Society
BCSA	*Burkholderia cepacia* Selective Agar
CF	Cystic Fibrosis
CFFPR	Cystic Fibrosis Foundation Patient Registry
CFFR	Cystic Fibrosis Foundation Registry
CFTR	Cystic Fibrosis Transmembrane Conductance Regulator
ECFSPR	European Cystic Fibrosis Society Patient Registry
ETI	Elexacaftor/Texacaftor/Ivacaftor
FEF25–75	Forced Expiratory Flow 25–75
FEV1	Forced Expiratory Volume in the 1st second
FVC	Forced Vital Capacity
IDSA	Infectious Diseases Society of America
MABSC	*Mycobacterium abscessus* Complex
MAC	*Mycobacterium avium* complex
MGIT	Mycobacterial Growth Indicator Tube
NTM	Nontuberculous Mycobacteria
NTM-PD	Nontuberculous Mycobacteria Pulmonary Disease
PEG	Percutaneous Endoscopic Gastrostomy
PRISMA	Preferred Reporting Items for Systematic Reviews and Meta-Analyses
RGM	Rapidly Growing Mycobacteria
U.K.	United Kingdom
U.S.A.	United States of America
